# Supplementary figures and images for: SARS-CoV-2 uses CD4 to infect T helper lymphocytes
Source: eLife. 2023 Jul 31;12:e84790. doi: 10.7554/eLife.84790 (PMC10390044; doi:10.7554/eLife.84790)

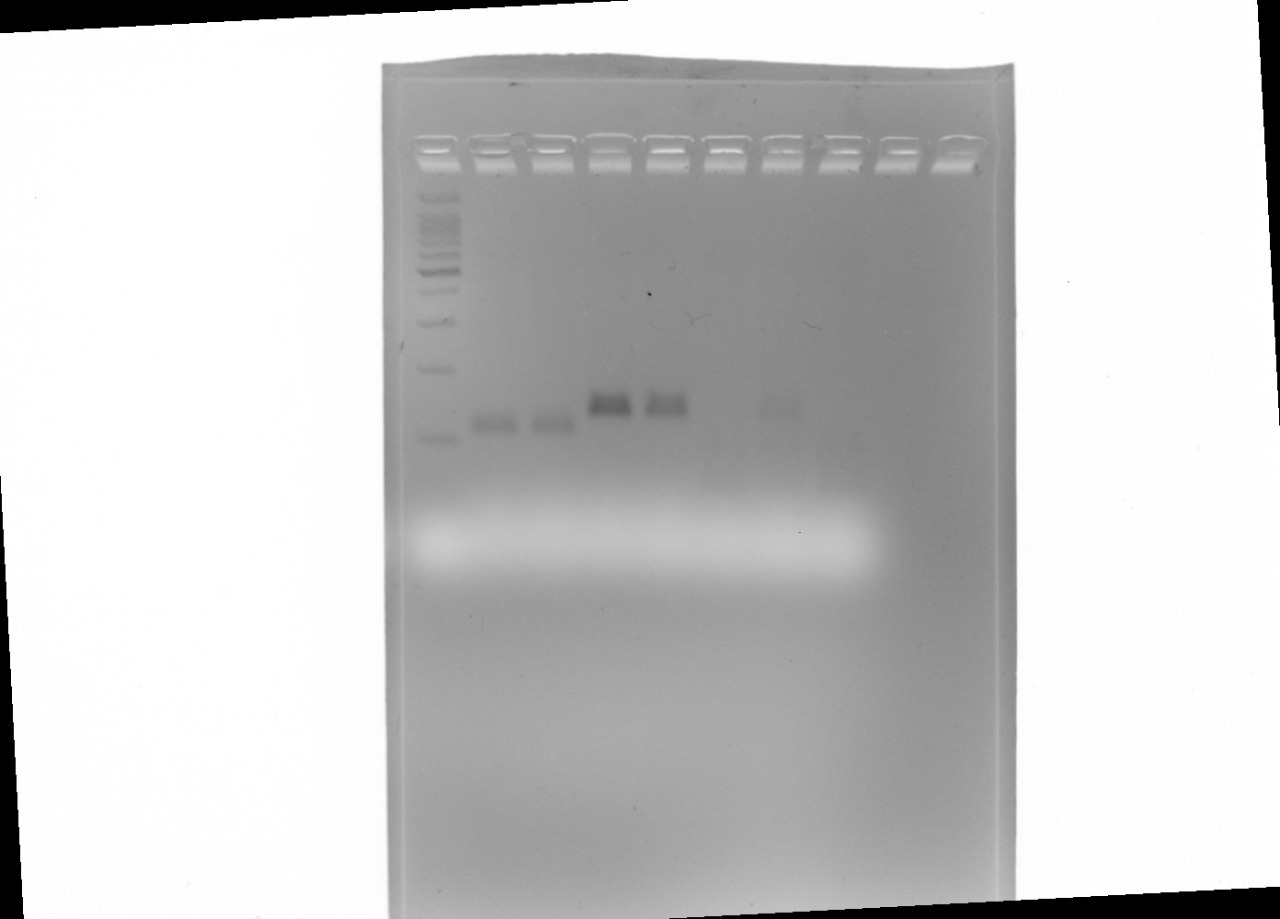

Supplement: Figure 1—figure supplement 4—source data 1. [file elife-84790-fig1-figsupp4-data1.zip › Figure 1- Figure Supplement 4- source data 1.jpeg]

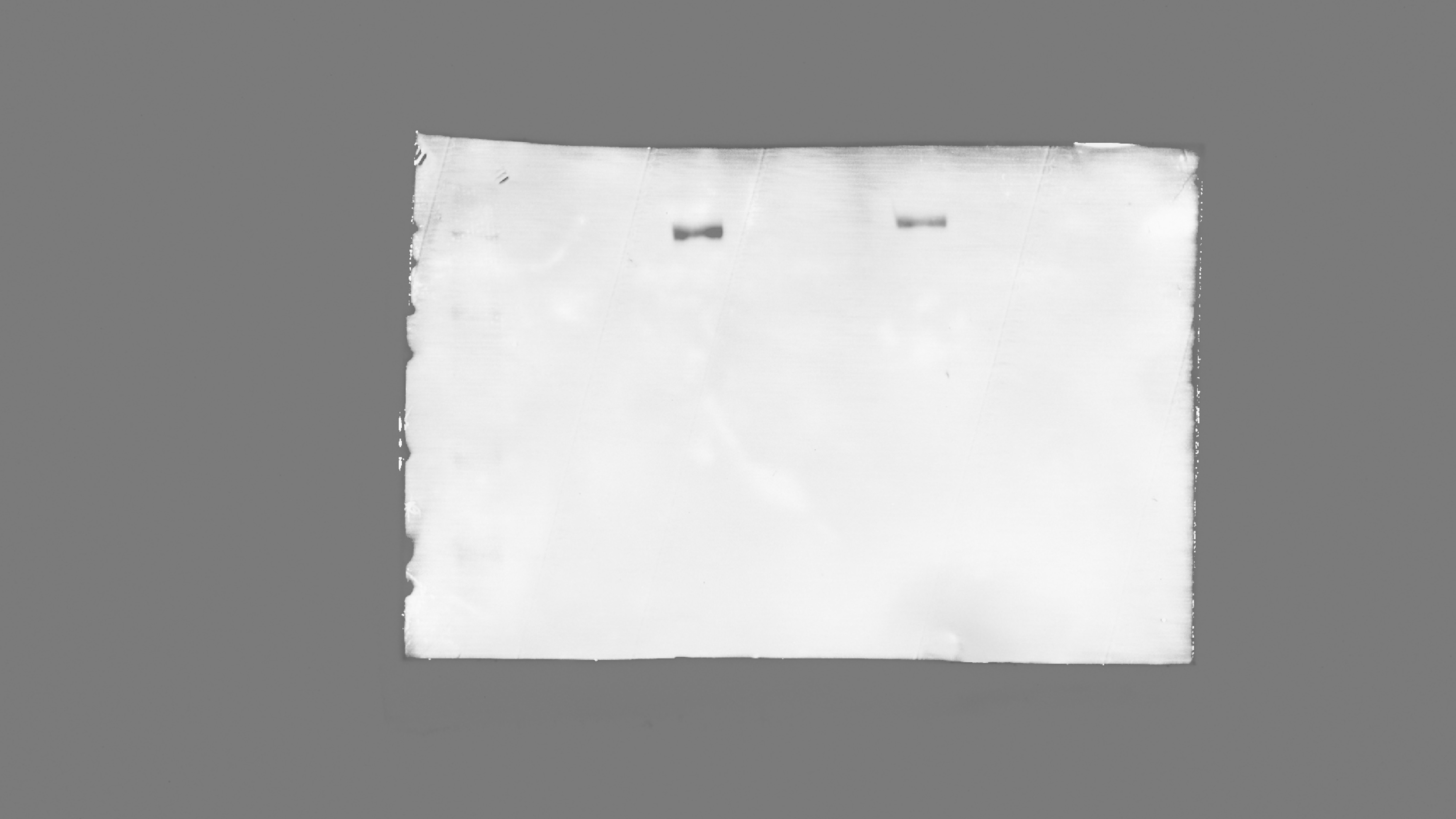

Supplement: Figure 3—source data 1. — Recombinant sCoV-2 and CD4 were co-incubated and immunoprecipitated with anti-CD4. [file elife-84790-fig3-data1.zip › Figure 3 - source data 1.jpg]

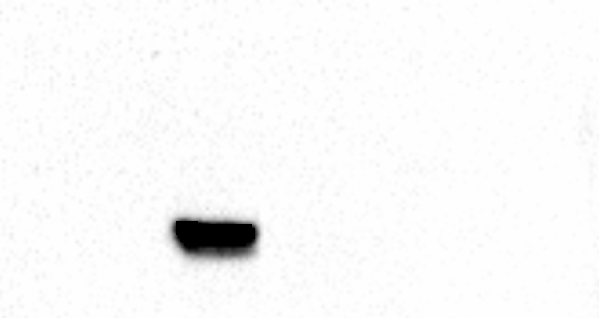

Supplement: Figure 3—source data 2. [file elife-84790-fig3-data2.zip › Figure 3- source data 2.tif]

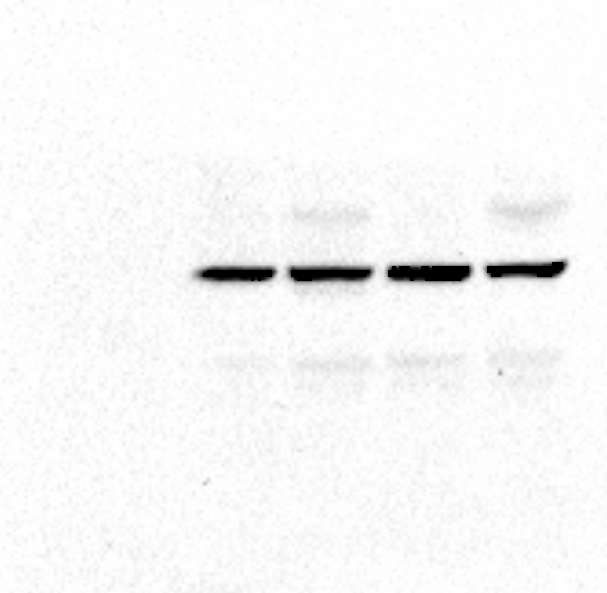

Supplement: Figure 3—source data 3. [file elife-84790-fig3-data3.zip › Figure 3- source data 3.tif]

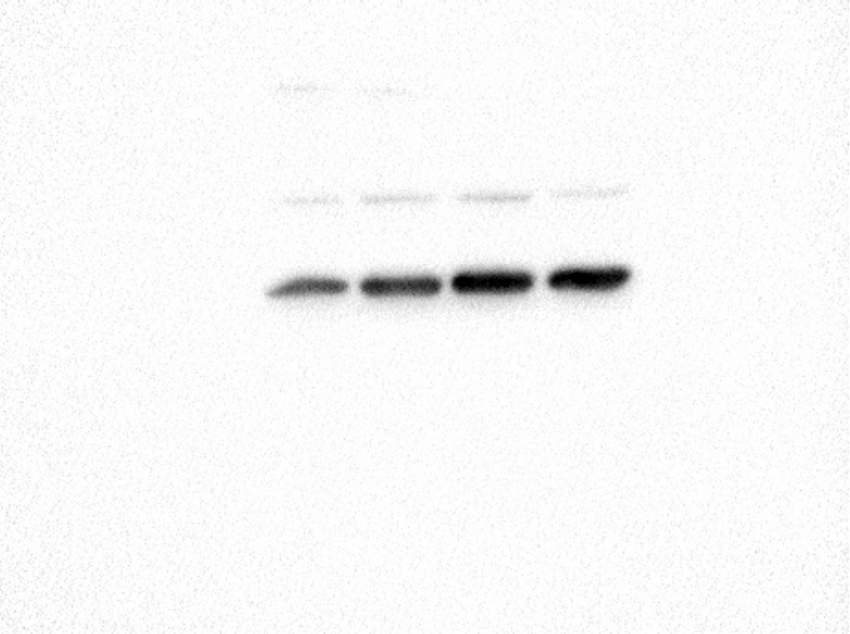

Supplement: Figure 3—source data 4. [file elife-84790-fig3-data4.zip › Figure 3- source data 4.tif]

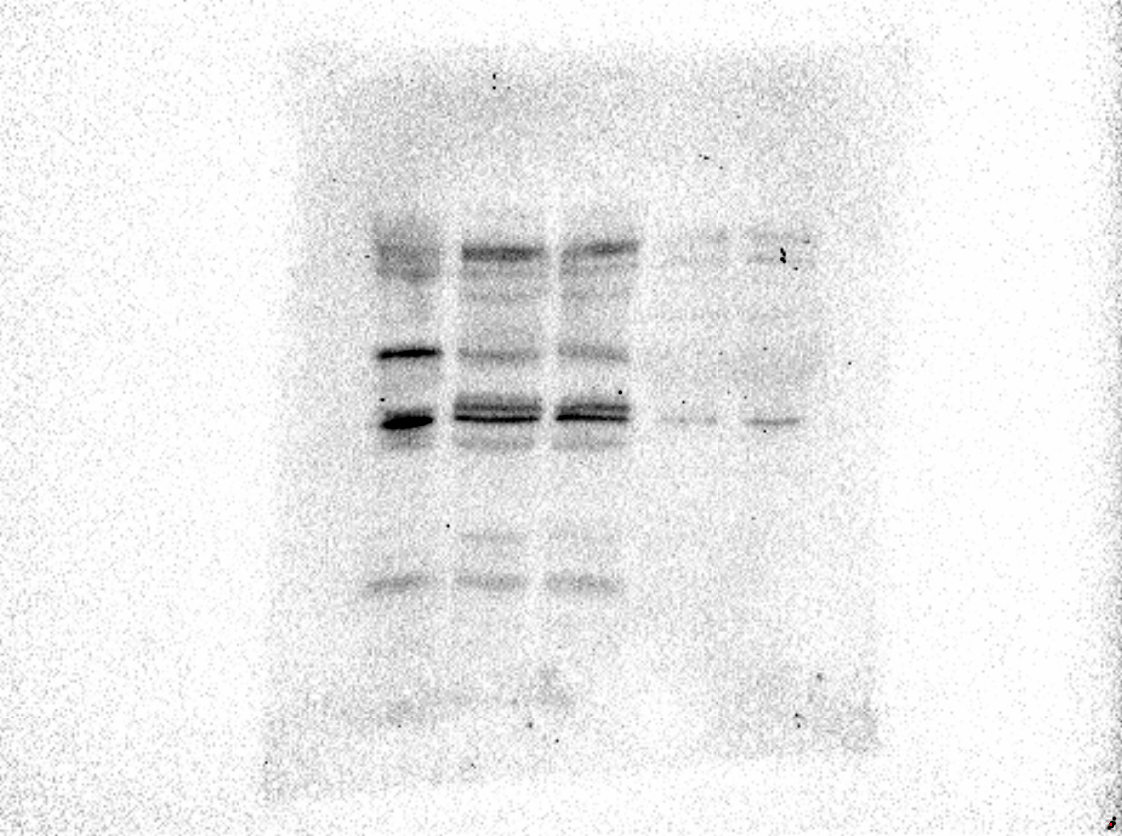

Supplement: Figure 3—figure supplement 1—source data 1. [file elife-84790-fig3-figsupp1-data1.zip › Figure 3- Figure supplement 1-source data 1.tif]

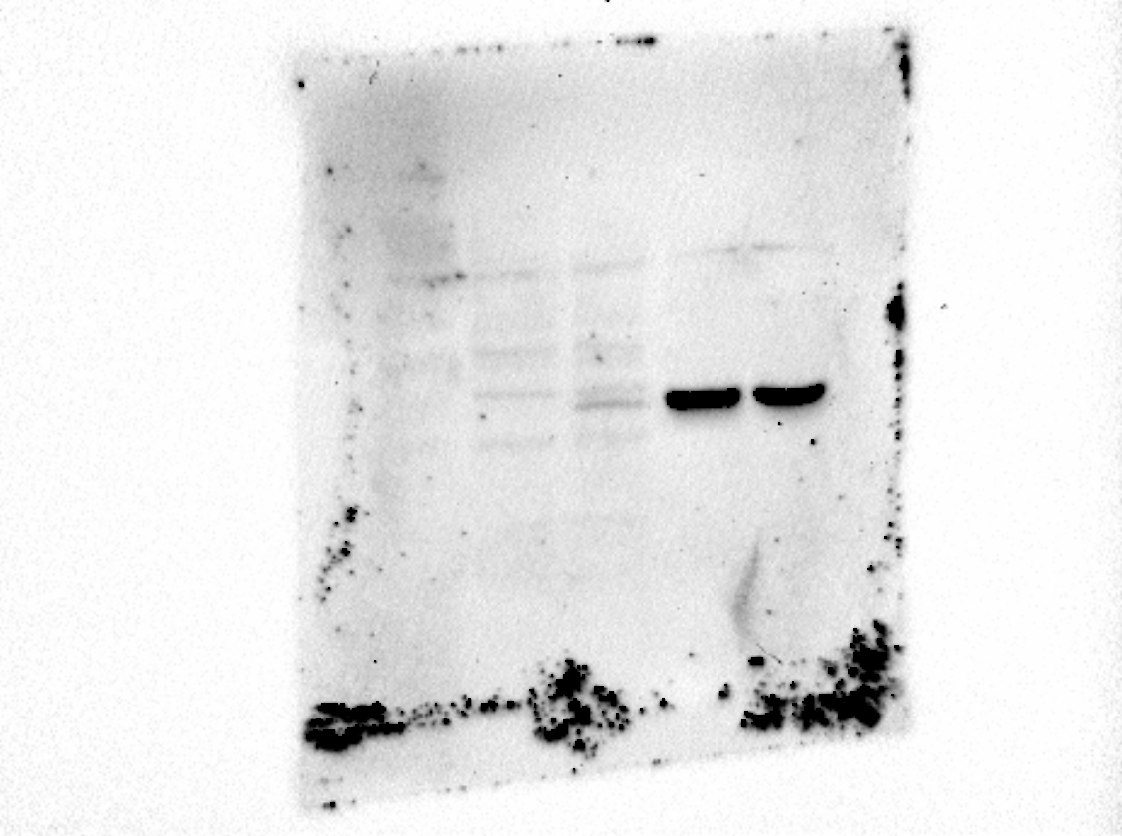

Supplement: Figure 3—figure supplement 1—source data 2. [file elife-84790-fig3-figsupp1-data2.zip › Figure 3- Figure Supplement 1- source data 2.tif]

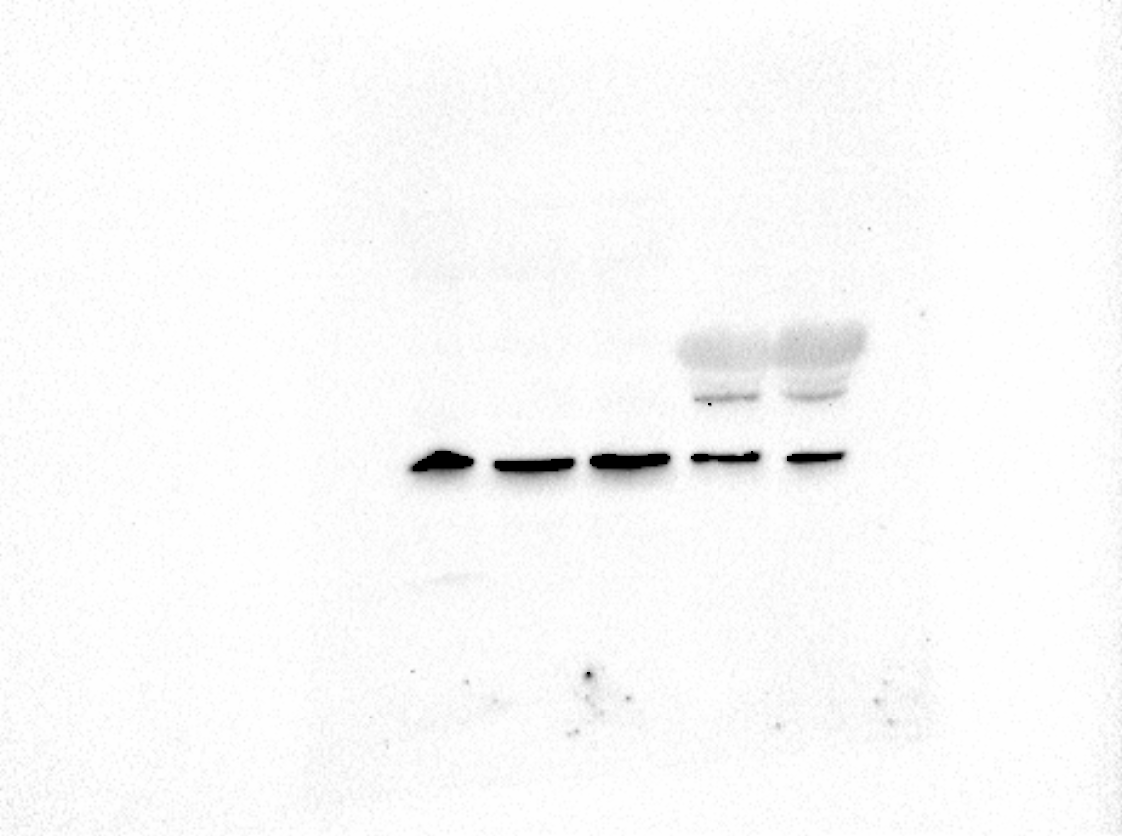

Supplement: Figure 3—figure supplement 1—source data 3. [file elife-84790-fig3-figsupp1-data3.zip › Figure 3- Figure supplement 1 - source data 3.tif]

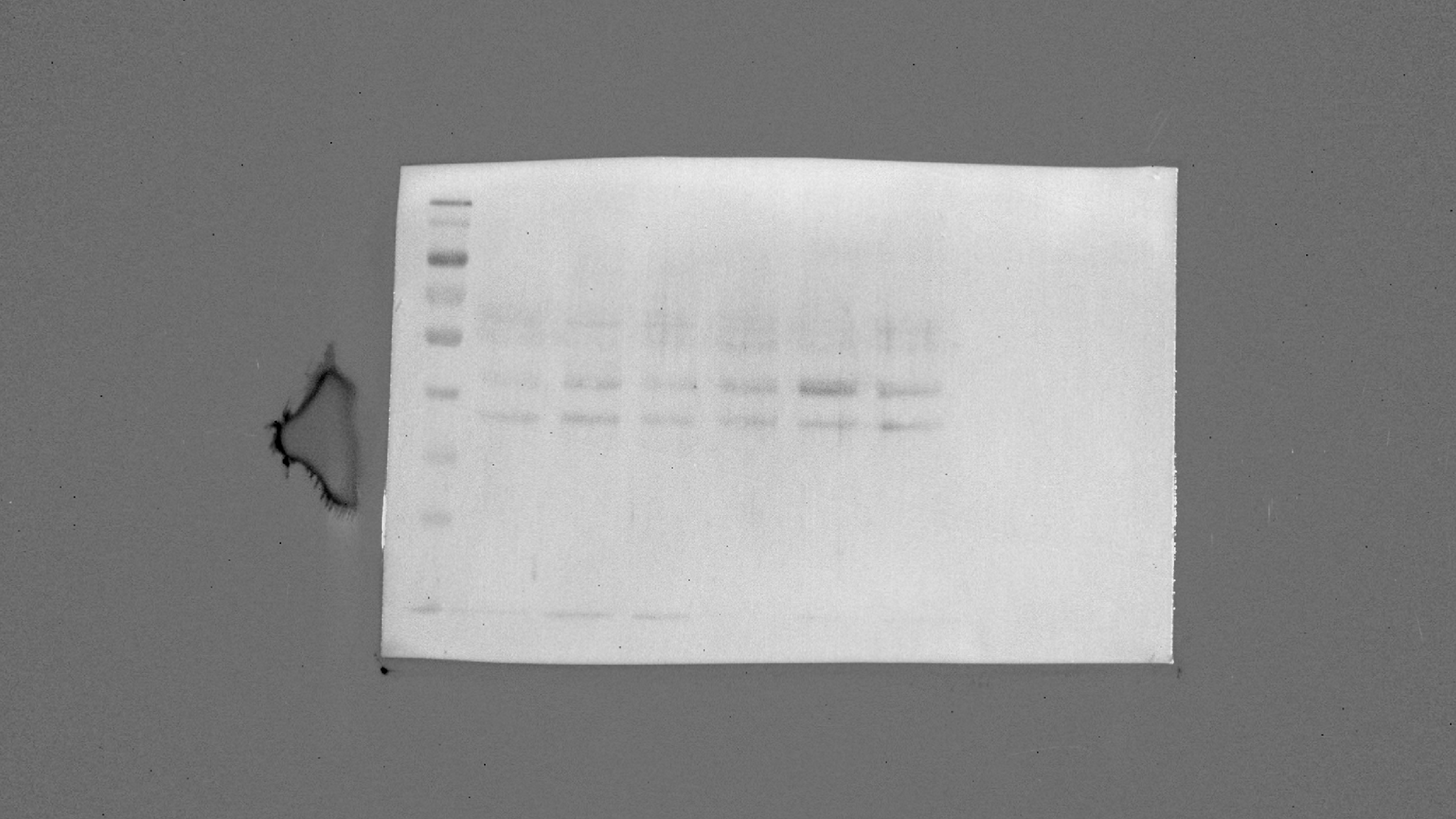

Supplement: Figure 4—source data 1. [file elife-84790-fig4-data1.zip › Figure 4 source data 1.jpg]

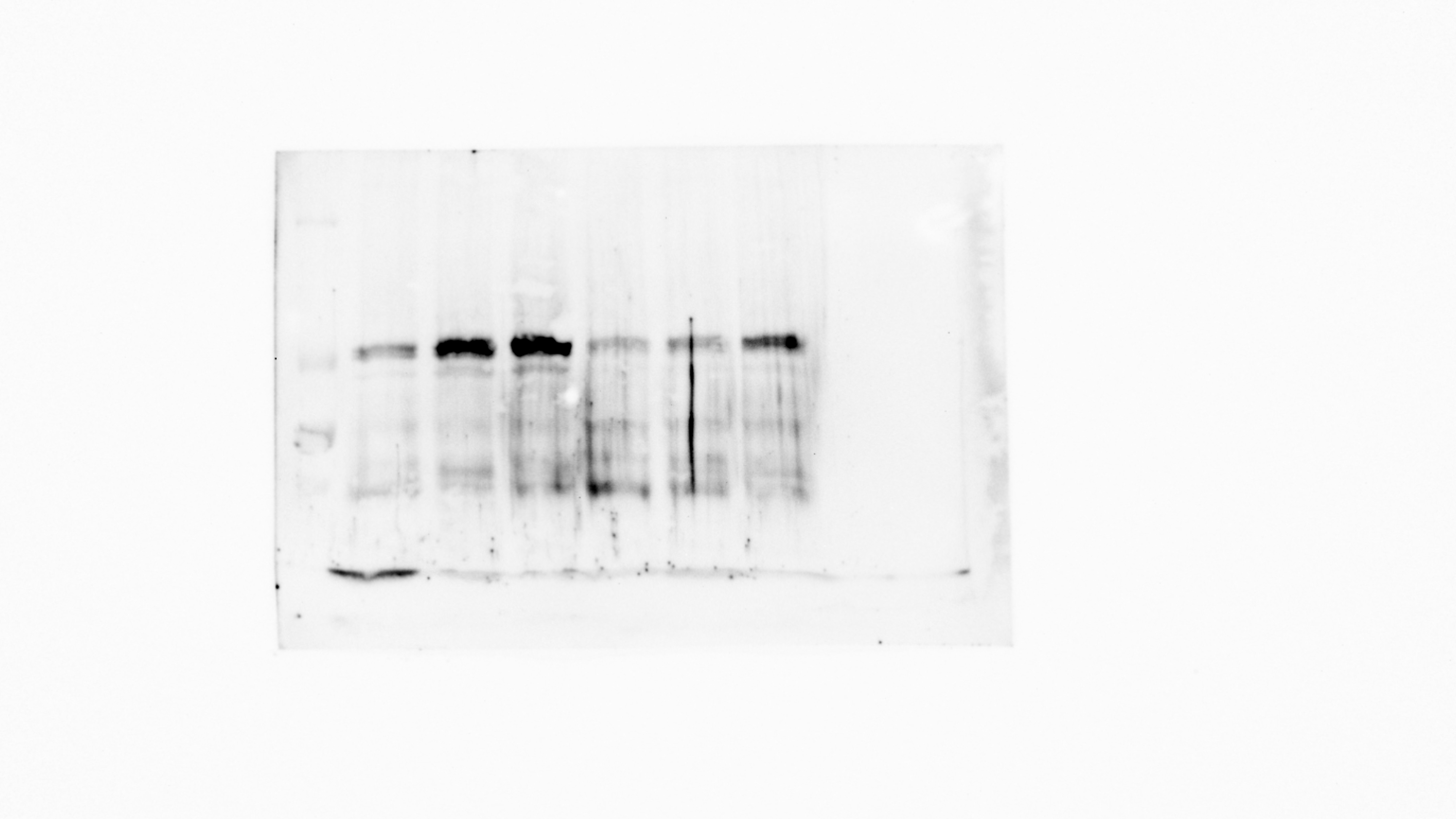

Supplement: Figure 4—source data 2. [file elife-84790-fig4-data2.zip › Figure 4- source data 2.jpg]

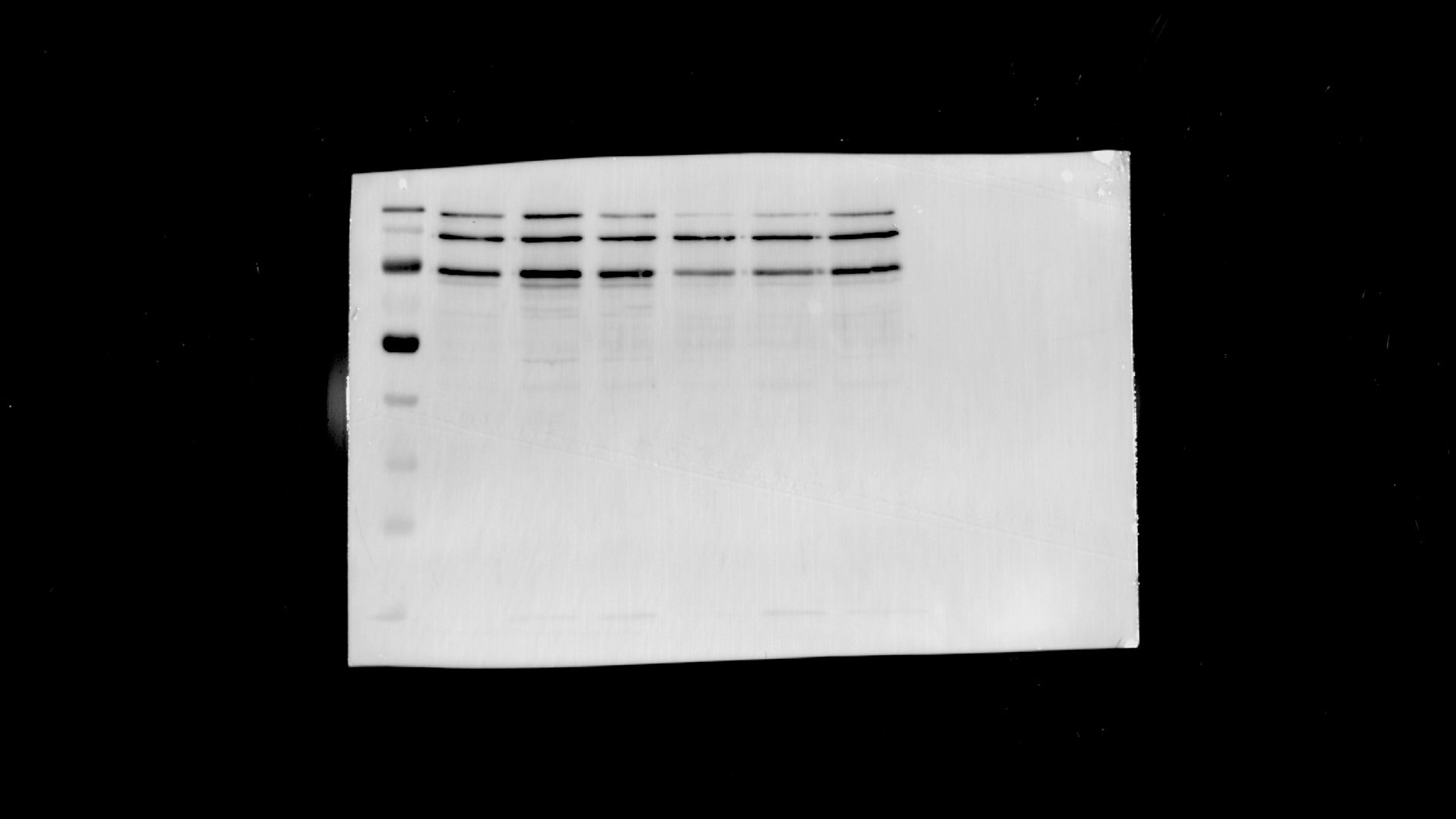

Supplement: Figure 4—source data 3. [file elife-84790-fig4-data3.zip › Figure 4- source data 3.jpg]

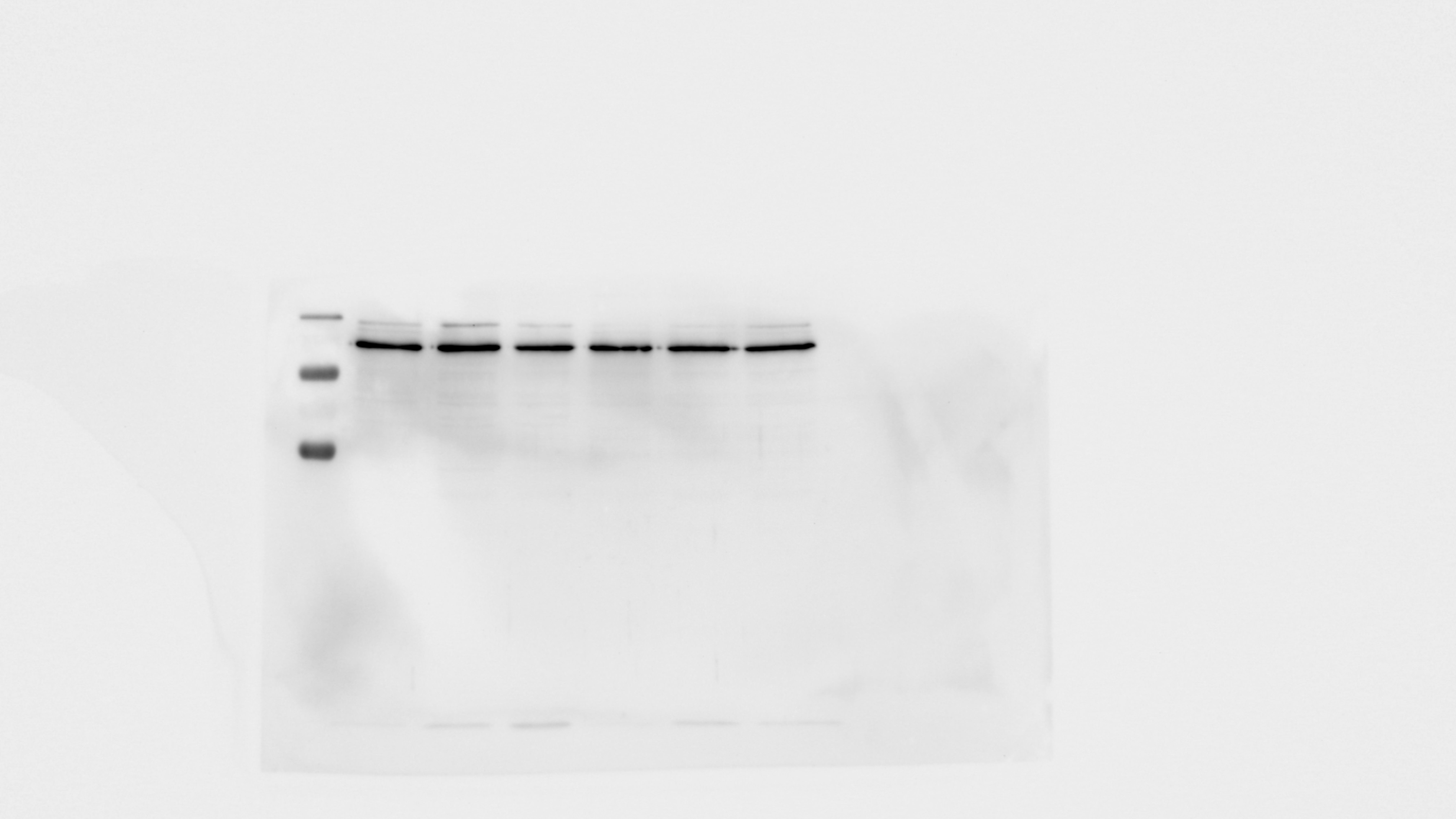

Supplement: Figure 4—source data 4. [file elife-84790-fig4-data4.zip › Figure 4- source data 4.jpg]
