# Supplementary material for: SARS-CoV-2 uses CD4 to infect T helper lymphocytes
Source: eLife. 2023 Jul 31;12:e84790. doi: 10.7554/eLife.84790 (PMC10390044; doi:10.7554/eLife.84790)
Supplement: Supplementary file 1. — Gender, age, and admittance oxygen saturation were evaluated. [file elife-84790-supp1.docx]

**Supplementary File 1**

| **Subject** | **Gender** | **Age range (y)**  **Mean (SD)** | **Admittance O2**  **saturation (%)**  **Mean (SD)** |
| --- | --- | --- | --- |
| **Healthy Donors** | | | |
| **H19 20 000001** | **M** | **26-30** | **-** |
| **H19 20 000002** | **M** | **41-45** | **-** |
| **H19 20 000003** | **M** | **46-50** | **-** |
| **H19 20 000004** | **F** | **51-55** | **-** |
| **H19 20 000005** | **M** | **41-45** | **-** |
| **H19 20 000006** | **F** | **36-40** | **-** |
| **H19 20 000007** | **M** | **31-35** | **-** |
| **H19 20 000008** | **F** | **26-30** | **-** |
| **H19 20 000009** | **M** | **46-50** | **-** |
|  | **M/F: 6:3** | **36.4 (9.60)** |  |
|  | | | |
| **COVID-19 Patients** | | | |
| **C19 20 000001** | **F** | **36-40** | **86 ra** |
| **C19 20 000002** | **F** | **41-45** | **91 ra** |
| **C19 20 000003** | **M** | **36-40** | **100 nrb** |
| **C19 20 000004** | **M** | **61-65** | **92 ra** |
| **C19 20 000005** | **F** | **41-45** | **89 ra** |
| **C19 20 000006** | **F** | **76-80** | **90 ra** |
| **C19 20 000007** | **F** | **56-60** | **92 ra** |
| **C19 20 000008** | **F** | **56-60** | **92 ra** |
| **C19 20 000009** | **F** | **56-60** | **94 ra** |
| **C19 20 000010** | **F** | **36-40** | **90 ra** |
| **C19 20 000011** | **M** | **41-45** | **87 ra** |
| **C19 20 000012** | **M** | **76-80** | **86 ra** |
| **C19 20 000013** | **F** | **41-45** | **98 ra** |
| **C19 20 000014** | **M** | **31-35** | **92 ra** |
| **C19 20 000015** | **M** | **36-40** | **93 ra** |
| **C19 20 000016** | **M** | **66-70** | **87 ra** |
|  | **M/F: 7:9** | **50.9 (15)** | **91.2 (3.9)** |
|  | | | |

***Patients IDs are not identifying in the manuscript.**
